# A Novel Technology Platform for Extracellular Vesicle-Targeted Expression of Drug-Metabolizing Enzymes: Driving CYP3A4 Expression and Secretion via the EABR Motif

**DOI:** 10.3390/biomedicines14061299

**Published:** 2026-06-08

**Authors:** Haihong Hu, Shaojun Zhou, Yi Peng, Yuru Liu, Zhiyuan Qin, Lushan Yu, Su Zeng

**Affiliations:** 1 Zhejiang Province Key Laboratory of Anti-Cancer Drug Research, State Key Laboratory of Advanced Drug Delivery and Release Systems, Institute of Drug Metabolism and Pharmaceutical Analysis, Research Center for Clinical Pharmacy, College of Pharmaceutical Sciences, Zhejiang University, Hangzhou 310058, China; 2 Department of Biology, School of Arts & Sciences, University of Pennsylvania, Philadelphia, PA 19104, USA

**Keywords:** Cytochrome P450 3A4, extracellular vesicles, ESCRT pathway, CEP55-EABR

## Abstract

**Background**: Cytochrome P450 3A4 (CYP3A4) is a key membrane-anchored drug-metabolizing enzyme. Its expression and purification in heterologous systems are severely hindered by low yield and detergent-induced structural inactivation. Although extracellular vesicles (EVs) provide an ideal natural lipid bilayer environment to stabilize membrane proteins, targeted loading remains challenging. The ESCRT and ALIX-binding region (EABR) of CEP55 can efficiently recruit core components of the endosomal sorting complex (ESCRT) to mediate membrane fission. **Objectives**: This study used the EABR motif to drive the targeted vesicular secretion of CYP3A4, thereby establishing a novel membrane protein engineering platform. **Methods and Results**: EABR was fused with fluorescent protein, confirming its specific mediation of vesicular secretion. Recombinant plasmids of EABR/CYP3A4 and its reverse mutant (R-EABR) were transfected into HEK293T cells. Western blot and midazolam-based metabolic assays showed that forward EABR significantly enhanced CYP3A4 expression and EV secretion, while R-EABR lost exocytosis function. EVs isolated by ultracentrifugation verified EABR’s role in recruiting ESCRT and improving CYP3A4 activity. **Conclusions**: Forward CEP55-EABR specifically and efficiently drives vesicular encapsulation of CYP3A4, enhancing its expression and secretion. This ESCRT-mediated strategy avoids destructive purification, provides a stable lipid-rich bioreactor for CYP3A4, and has great translational potential in high-throughput in vitro drug metabolism and screening platforms.

## 1. Introduction

Drug-metabolizing enzymes are core molecular machines mediating the biotransformation of xenobiotics. Among mammalian Phase I metabolic enzymes, the cytochrome P450 (CYP) superfamily has consistently been a focal point in pharmacokinetics and pharmacology due to its critical role in the metabolism of clinical drugs, steroids, and endogenous lipids [1]. Notably, CYP3A4 is the most abundant and vital isoform, predominantly expressed in the liver and small intestine [2], and CYP3A family members are responsible for the metabolic clearance of approximately 50% of small-molecule drugs in clinical use [3,4]. As a typical membrane-anchored protein, CYP3A4 is anchored to the endoplasmic reticulum membrane via a highly hydrophobic N-terminal transmembrane helix, with its catalytic domain partially embedded in the lipid bilayer [5,6]. In studies of enzyme activity and drug–drug interactions (DDIs), midazolam (MDZ) is widely employed as a classic, specific probe substrate. Its metabolism exhibits a distinct concentration-dependent dual-pathway characteristic (yielding 1′-OH-MDZ and 4-OH-MDZ) [7].

This strict membrane dependence poses significant challenges for in vitro mechanistic studies [8]. Traditionally, heterologous expression of cytochrome P450 enzymes has heavily relied on bacterial (e.g., *Escherichia coli*) [9] and yeast systems [10]. While these systems offer rapid growth and ease of genetic manipulation, they fundamentally lack the native mammalian lipid environment and often fail to perform correct post-translational modifications. This frequently results in suboptimal membrane protein folding, rapid aggregation, or significantly impaired catalytic activity, particularly for highly hydrophobic, membrane-anchored human enzymes like CYP3A4. Thus, there is an urgent need for a mammalian-cell-derived platform that can provide a native-like lipid microenvironment to preserve the structural and functional integrity of these complex metabolic enzymes. The expression level of native CYP3A4 in heterologous hosts is notoriously low, and the obligatory use of detergents during traditional extraction and purification processes frequently disrupts its conformation, leading to an irreversible loss of enzymatic activity (conversion to the inactive P420 state) [11]. Although recent advancements in Nanodisc technology have provided a biomimetic lipid environment by in vitro reconstitution of phospholipid bilayers [12,13,14], which has been shown to significantly influence substrate binding and enzyme kinetics, this technique still relies on laborious in vitro assembly steps and the addition of exogenous membrane scaffold proteins (MSPs) [15]. Therefore, developing a novel membrane protein engineering strategy that enables spontaneous intracellular assembly, circumvents detergent-induced damage, and achieves the efficient secretion of single drug-metabolizing enzymes has emerged as a crucial methodological frontier to overcome current bottlenecks.

Extracellular vesicles (EVs) are natural lipid bilayer-enclosed entities with diameters ranging from 50 to 150 nm. Serving as essential vehicles for intercellular communication, EVs can encapsulate specific proteins and nucleic acids for extracellular secretion via budding. Topologically, this process requires the plasma membrane or multivesicular body (MVB) membrane to deform and undergo fission away from the cytoplasm. The core driving force for this event originates from the Endosomal Sorting Complex Required for Transport (ESCRT) machinery [16]. The ESCRT system comprises multiple subunits and accessory proteins (e.g., ALIX [17], VPS4 [18]). Within this system, the EABR (ESCRT- and ALIX-binding region) of the centrosomal protein CEP55 acts as a molecular hinge, recruiting the core ESCRT-I member TSG101 and ALIX with high affinity to efficiently mediate membrane fission and vesicle release [19,20]. Notably, numerous enveloped viruses (such as HIV-1 [21] and Ebola virus [22]) harness specific motifs on their structural proteins to “hijack” the host’s ESCRT machinery (including TSG101^8^ and ALIX [23]), thereby driving the efficient budding and release of virions from the host cell membrane.

The mechanism by which viruses hijack the ESCRT pathway offers a highly inspiring technological route for the engineered in vitro expression and delivery of complex membrane proteins. Recent studies have demonstrated that fusing specific recruitment motifs, such as CEP55-EABR, to the intracellular terminus of exogenous membrane proteins can artificially redirect the ESCRT machinery. For instance, the introduction of the EABR motif into the cytoplasmic tail of the SARS-CoV-2 spike protein increased the vesicular secretion of the target protein in non-immune cells 10- to 100-fold, successfully inducing the efficient assembly and budding of enveloped virus-like particles (eVLPs) [24]. Most remarkably, a recent breakthrough study demonstrated that the EABR tag can serve as a versatile membrane protein engineering tool to efficiently target full-length human transmembrane receptors (e.g., epidermal growth factor receptor, EGFR) into extracellular vesicles (EVs) [25]. Crucially, the native membrane environment of these EVs perfectly preserved the 3D active conformation and assembly characteristics of the receptors. Building upon this background, the present study aims to utilize the CEP55-EABR motif to drive the vesicular secretion of CYP3A4, generating a highly stable, lipid-rich native bioreactor via intracellular self-assembly, thereby providing a novel engineering paradigm for the in vitro study of drug-metabolizing enzymes.

Based on the above research background, this study proposes a novel technical strategy for driving the budding of membrane-anchored drug-metabolizing enzymes using the ESCRT pathway. We fused the CEP55-EABR motif with CYP3A4, with the core purpose of hijacking the endogenous cellular ESCRT machinery to achieve direct sorting, encapsulation, and secretion of the membrane-anchored protein CYP3A4 into extracellular vesicles (EVs). During the research process, we first verified that the EABR element can specifically mediate the vesicular secretion of the target protein by inserting a fluorescent protein; subsequently, using an experimental system with forward and reverse insertion of the EABR element, we further confirmed that this secretion pathway has strict sequence specificity; on this basis, extracellular vesicles were isolated through experiments, and the expression level and metabolic activity of CYP3A4 in the vesicles were systematically verified. The results showed that not only can the natural vesicularization driven by EABR significantly improve the expression level and catalytic activity of CYP3A4, but also the natural lipid membrane microenvironment of EVs can provide excellent in vitro structural and functional stability for the enzyme. The results obtained in this study will provide a new technical platform for the efficient and stable in vitro expression of drug-metabolizing enzymes, and show great translational application potential in the fields of membrane protein engineering and high-throughput drug screening.

## 2. Materials and Methods

### 2.1. Construction of Recombinant Expression Vectors for CYP3A4

EABR and EPM were amplified separately with primer pairs 5/3-EABR and 5/3-EPM, then fused into EABR-EPM by SOE-PCR. The EABR-EPM sequence was inserted at the N-terminus of CYP3A4 and amplified from human cDNA using specific primers (5N/3N-CYP3A4). For insertion at the C-terminus, primers (5C/3C-CYP3A4) were used. For reverse insertion of the EABR sequence, amplification was performed with primers 5R/3R-CYP3A4. All primer sequences mentioned above are listed in Supplementary Table S1. NheI and ApaI sites were added to CYP3A4 primers; ApaI and BamHI sites to EABR-EPM primers. The pcDNA3.1-signal-sfGFP-EABR vector was double-digested with NheI/BamHI (Thermo Fisher Scientific, Waltham, MA, USA) (37 °C, 15 min) and gel-purified. Following double restriction digestion, a ~5.4 kb vector backbone fragment was obtained. The corresponding schematic vector maps are detailed in Supplementary Figure S1.

The three fragments (CYP3A4, EABR-EPM, linearized vector) were mixed at 3:3:1 molar ratio with 1 μL seamless cloning enzyme (Comwin, Beijing, China) and 2 μL buffer (10 μL total), incubated at 50 °C for 30 min, then 4 °C for 3 min. The product was transformed into TOP10 competent cells (ice 30 min, 42 °C 90 s, ice 5 min), recovered in 1 mL LB medium at 37 °C for 1 h, and plated overnight. Single colonies were cultured for 6 h, and PCR with CYP3A4 primers confirmed the insert by agarose gel electrophoresis.

### 2.2. Cell Culture and Plasmid Transfection

HEK293T cells were cultured in Dulbecco’s modified Eagle’s medium (Corning, Shanghai, China)with 10% FBS (Sigma-Aldrich, St. Louis, MO, USA) and 1% penicillin-streptomycin (Servicebio, Wuhan, China) at 37 °C in 5% CO_2_. For transfection, cells were seeded one day prior. In a 24-well plate, 500 ng plasmid DNA was mixed with 0.5 μL jetPRIME (Polyplus, Shanghai, China) in 50 μL buffer, incubated 10 min at RT, and added dropwise to cells. After 6 h at 37 °C, medium was replaced with 3% FBS medium, and cells were incubated for 72 h or 96 h. For a 10 cm dish, all volumes were scaled 20-fold (1 mL transfection mixture). The cell transfection efficiency was evaluated to be approximately 70–80%.

### 2.3. Protein Extraction and Western Blot Analysis

After transfection, cell culture supernatant was collected. Then, 40 µL of supernatant was mixed with 40 µL RIPA buffer (Beyotime, Shanghai, China) and 20 µL 5× loading buffer (Sangon, Shanghai, China) and heated at 100 °C for 10 min. Adherent cells were washed with ice-cold PBS, lysed in RIPA buffer containing 1% PMSF (Sangon, Shanghai, China) (150 µL/well) on ice for 30 min, and centrifuged at 13,000× *g* for 10 min at 4 °C. The supernatant (80 µL) was mixed with 20 µL 5× loading buffer and heated at 100 °C for 10 min. Protein samples (30 µg/lane) were separated on a 10% SDS-PAGE gel (80 V through stacking gel, then 120 V through resolving gel) and transferred onto a methanol-activated PVDF membrane (Sigma-Aldrich, St. Louis, MO, USA) at 400 mA for 30 min. The membrane was blocked with 5% non-fat milk in TBST for 2 h at room temperature, washed three times with TBST, and incubated overnight at 4 °C with anti-CYP3A4 (Cat# 18227-1-AP, Proteintech, Wuhan, China) (1:2000) and anti-GAPDH (Cat# 60004-1-Ig, Proteintech, Wuhan, China) (1:5000). After washing, the membrane was incubated with HRP-conjugated secondary antibodies (Cat# RGAR001, Proteintech, Wuhan, China) (1:5000) for 1 h at room temperature. Protein bands were visualized using an ECL kit (Vazyme, Nanjing, China) and a chemiluminescence imaging system (Azure Biosystems, Dublin, CA, USA). For quantitative analysis, the optical densities of the protein bands were analyzed using ImageJ 1.46r software (NIH). The relative expression level of the target protein was calculated by normalizing its absolute band intensity to that of the corresponding internal control (e.g., GAPDH). The final normalized values were expressed as fold changes relative to the designated control group.

### 2.4. Substrate Incubation for CYP3A4

At 48 h post-transfection, the original culture medium was aspirated, and 200 μL/well of HBSS buffer containing MDZ (1 μg/mL) pre-warmed to 37 °C was added. The cells were incubated at 37 °C for 3 h. After incubation, the supernatant was collected, and 200 μL/well of 0.1% SDS solution was added to lyse the remaining cells. Appropriate aliquots of the cell lysate and supernatant samples were mixed with methanol containing the internal standard loratadine (Sigma-Aldrich, St. Louis, MO, USA) (1:1000 dilution) at a volume ratio of 1:3 to quench the reaction and precipitate proteins. After vortexing, the mixture was centrifuged at 13,000× *g* for 10 min at 4 °C, and the supernatant was collected for subsequent LC-MS/MS analysis. The relative 1′-OH-MDZ production was determined by LC-MS/MS, utilizing the peak area ratio of the metabolite to the internal standard (loratadine).

### 2.5. Liquid Chromatography–Tandem Mass Spectrometry Analysis

Quantification of MDZ metabolites was performed using liquid chromatography–tandem mass spectrometry (LC-MS/MS) (Agilent, Santa Clara, CA, USA). The analysis was conducted on an Agilent 1290-6460 triple quadrupole mass spectrometer, and the ratio of the chromatographic peak area of 1′-OH MDZ to that of the internal standard loratadine was used as the activity evaluation index.

Chromatographic conditions: An Agilent Eclipse XDB-C18 column (Agilent, Santa Clara, CA, USA) (100 mm × 2.1 mm, 3.5 μm) was used at a column temperature of 30 °C. Mobile phase A was 0.1% formic acid in water, and mobile phase B was neat methanol. The flow rate was 0.3 mL/min, and the injection volume was 10 μL. The gradient elution program was as follows: 0–0.2 min, 30% B; 0.2–1.5 min, B linearly increased to 95%; 1.5–2.8 min, held at 95% B; 2.8–2.81 min, B decreased to 30%; 2.81–3.5 min, re-equilibrated at 30% B. The total run time was 3.5 min.

Mass spectrometric conditions: Electrospray ionization (ESI) in positive ion mode was employed, and detection was performed in multiple reaction monitoring (MRM) mode. The ion source parameters were as follows: capillary voltage, 3500 V; cone voltage, 500 V; drying gas temperature, 350 °C; nebulizer pressure, 45 psi; auxiliary gas pressure, 45 psi; curtain gas pressure, 15 psi. The MRM transitions were *m*/*z* 342.01 → 324.1 for 1′-OH MDZ and *m*/*z* 383.1 → 337.1 for loratadine.

### 2.6. Isolation and Characterization of Extracellular Vesicles from Cells

At 96 h post-transfection, the cell culture supernatant from 10 cm dishes was collected, and extracellular vesicles (EVs) were isolated by differential centrifugation. First, the supernatant was centrifuged at 4000 rpm for 30 min at 4 °C, and this step was repeated twice to remove cell debris. The supernatant was then filtered through a 0.22 μm PES membrane filter. The filtrate was transferred to an ultracentrifuge tube and subjected to ultracentrifugation at 150,000× *g* for 2 h at 4 °C to pellet the vesicles using the Beckman Coulter Optima XE centrifuge (Beckman Coulter, Brea, CA, USA). After carefully discarding the supernatant, the vesicle pellet was resuspended in 200 μL of sterile PBS, appropriately diluted, aliquoted, and stored at −80 °C until use.

The resistive pulse sensing (RPS) strategy has been extensively exploited to determine the size and concentration of EVs, as described in [26,27]. The size distribution and the concentration of the isolated EVs were determined using a NanoCoulter counter (Resun Technology, Co., Ltd., Shenzhen, China) with a custom chip with a measuring range suited for detecting the analyte, and vesicle morphology was examined via Talos L 120 C transmission electron microscopy (Thermo Fisher Scientific, Waltham, MA, USA). EVs were diluted 20-fold in PBS prior to RPS analysis.

For Western blot analysis of EV markers, the sample preparation, electrophoresis, and membrane transfer were performed as described in Section 2.3. Antibodies against ALIX, CD63, and TSG101 were used as positive EV markers, and an antibody against the endoplasmic reticulum-resident protein calnexin was strictly included as a negative control to assess EV purity. Anti-ALIX (Cat# ET1705-74), anti-CD63 (Cat# ET1607-2), anti-TSG101 (Cat# ET1701-59), and anti-calnexin (Cat# ET1611-86) (HUABIO, Hangzhou, China) were rabbit monoclonal antibodies used at a dilution of 1:2000.

### 2.7. P450-Glo™ CYP3A4 Assay

The assay was performed following the protocol of the P450-Glo™ CYP3A4 Assay (Promega, Beijing, China) with Luciferin-IPA for Cell-Based Samples. In this assay, Luciferin-IPA serves as a specific pro-luminescent substrate for CYP3A4. CYP3A4 specifically cleaves Luciferin-IPA to release free D-luciferin, which subsequently reacts with excess exogenous luciferase provided in the detection reagent to generate luminescence. Therefore, the measured relative luminescence units (RLU) exclusively quantify the catalytic turnover of CYP3A4, ruling out any interference from endogenous luciferase expression. First, the 3 μM Luciferin-IPA stock solution was diluted 200-fold with PBS to a final concentration of 15 nM. Then, 20 μL of cell lysate or EV vesicle sample was mixed with 5 μL of the 15 nM Luciferin-IPA solution, followed by the addition of 25 μL of Detection Reagent. After mixing, the reaction mixture was incubated at room temperature for 20 min, and the luminescence signal was measured using a luminometer (Promega, Beijing, China). Total cellular protein was quantified using the BCA method.

### 2.8. Statistical Analysis

Experiments were carried out in duplicate or triplicate and data were expressed as mean ± SEM. Immunofluorescence images were quantified using ImageJ. Imaging data were analyzed by Student’s *t*-test. A *p*-value < 0.05 was considered statistically significant.

## 3. Results

### 3.1. Functional Verification of EABR-Mediated Targeted Vesicular Secretion

Recent studies have demonstrated that the ESCRT- and ALIX-binding region (EABR) derived from the human centrosomal protein CEP55 specifically recruits host ESCRT core components, such as TSG101 and ALIX. When fused to the cytoplasmic tail of a target protein alongside an endocytosis prevention motif (EPM)—which extends the plasma membrane residency time of the chimera—the EABR module strongly drives the budding, assembly, and robust release of enveloped virus-like particles (eVLPs) or extracellular vesicles (EVs) presenting the target protein.

To visually confirm the capability of the EABR motif to drive vesicular secretion in our system, we engineered three superfolder green fluorescent protein (sfGFP)-based eukaryotic expression plasmids: (1) pcDNA3.1-sfGFP, serving as an intracellular control expressing cytosolic sfGFP; (2) pcDNA3.1-signal-sfGFP, incorporating a conventional signal peptide to mediate soluble secretion; and (3) pcDNA3.1-signal-sfGFP-EABR, the experimental construct fusing both the signal peptide and the EABR motif. These plasmids were individually transfected into HEK293T cells, and the fluorescence distribution within the cells and their conditioned media was evaluated by fluorescence microscopy at 48 h post-transfection.

**Figure 1:**
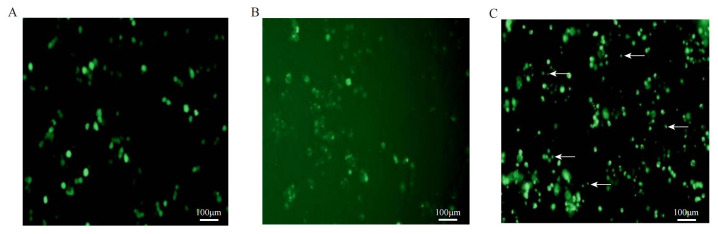
Fluorescence microscopy analysis of EABR-mediated vesicular secretion. Cells and conditioned media were imaged 48 h post-transfection using an Nikon Tis fluorescence microscope (Nikon Corporation, Melville, NY, USA) (equipped with a 10× objective). The images shown are representative fields of vision, serving as a qualitative morphological proof-of-concept for targeted vesicular secretion. Scale bars = 100 μm. (**A**) pcDNA3.1-sfGFP; (**B**) pcDNA3.1-signal-sfGFP; (**C**) pcDNA3.1-signal-sfGFP-EABR.

As shown in Figure 1, cells expressing the pcDNA3.1-sfGFP control exhibited a uniform green fluorescent signal strictly confined to the cytoplasm, with no detectable fluorescence in the surrounding medium. In contrast, cells transfected with pcDNA3.1-signal-sfGFP displayed a pronounced, diffuse green fluorescent background in the culture medium, confirming that sfGFP was secreted extracellularly as a soluble protein. Strikingly, in the condition expressing the EABR-fused construct, the extracellular medium lacked a diffuse fluorescent background; instead, the signal appeared as abundant, highly concentrated particulate fluorescent puncta. This morphological distinction indicates that the EABR motif not only promotes the extracellular export of the target protein, but specifically alters its secretion modality—redirecting it from a free, soluble state into a dense, vesicle-encapsulated form. While these fluorescence observations served as an initial qualitative proof-of-concept, the rigorous biophysical and biochemical quantification of these vesicles was subsequently performed via RPS, TEM, and Western blotting (see Section 3.5). Encouraged by this robust vesicular sorting capability, we next investigated whether this EABR-driven EV engineering strategy could be applied to overcome the notoriously low expression and rapid inactivation of complex membrane-anchored drug-metabolizing enzymes, specifically CYP3A4.

### 3.2. Topological Design and Construction of CYP3A4-EABR Expression Plasmids

CYP3A4 possesses a highly specific topological architecture, comprising a highly hydrophobic N-terminal transmembrane (TM) helix that anchors the protein to the endoplasmic reticulum or plasma membrane, and a massive cytosol-facing globular catalytic domain containing the iron-heme prosthetic group. The amino acid sequence of CYP3A4 is shown in Figure S2. This unique membrane-anchored configuration allows the catalytic domain to partially embed into the lipid bilayer, facilitating the direct recruitment and recognition of lipophilic substrates from the membrane phase for subsequent oxidative catalysis.

**Figure 2:**
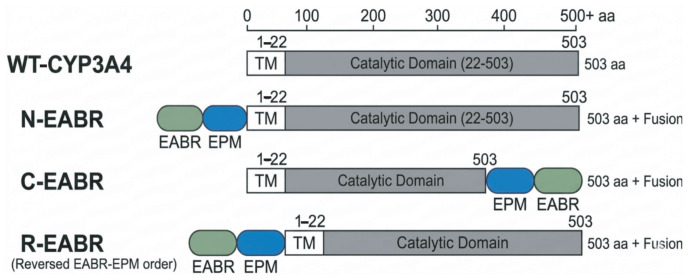
Schematic diagram of different CYP3A4 construct structures.

As demonstrated in Section 3.1, the CEP55-EABR motif can efficiently induce membrane fission and promote the vesicular secretion of target proteins by recruiting cytosolic components of the ESCRT machinery (e.g., TSG101 and ALIX). Because ESCRT-driven membrane budding strictly occurs on the cytosolic face of the membrane, the spatial topology of the EABR motif relative to the target protein’s TM anchor is hypothesized to be a critical determinant of vesicular sorting efficiency. To systematically investigate this topological dependence, we designed a series of CYP3A4 fusion expression vectors with varying configurations (Figure 2). Specifically, the EABR motif was fused to either the N-terminus (N-EABR-CYP3A4) or the C-terminus (C-EABR-CYP3A4) of CYP3A4. Furthermore, to prevent rapid internalization of the chimeric proteins and extend their residency time at the plasma membrane—thereby providing an adequate time window for ESCRT recruitment and budding assembly—an endocytosis prevention motif (EPM) was fused in tandem with the EABR sequence.

Crucially, to rigorously verify whether the vesicular exocytosis of CYP3A4 is strictly dependent on the specific spatial recognition of the ESCRT machinery by the EABR structure, we designed a mutant vector featuring a mirrored (retro-inverso) EABR amino acid sequence (R-EABR-CYP3A4). This reverse sequence insertion completely abolishes the canonical parallel coiled-coil structure of EABR, serving as an impeccable structural negative control while maintaining the exact identical molecular weight and amino acid composition. To computationally elucidate the structural basis for the functional abrogation of the reversed construct, we performed AlphaFold-Multimer modeling alongside single-chain confidence profiling and sequence-based heptad fingerprint analysis (Supplementary Figure S3). For the forward wild-type chimera (EPM-EABR), the model predicted a highly confident and specific parallel coiled-coil dimeric assembly, evidenced by high interface confidence scores (ipTM ≈ 0.56; ipSAE ≈ 0.565) and a modest Mean Predicted Aligned Error (Mean PAE ≈ 18.22). In stark contrast, the reversed chimera (EABR-EPM) exhibited a catastrophic loss of interface confidence and specificity (ipTM ≈ 0.21; ipSAE ≈ 0.012) alongside uniformly elevated inter-chain PAE (Mean PAE ≈ 22.86). Our analysis reveals that this profound structural failure is fundamentally driven by two synergistic mechanisms. First, sequence-based heptad scanning demonstrated that sequence reversal causes a massive shift in the optimal heptad phase (from r = 1 to r = 6). This shift mathematically destroys the geometric complementarity of the canonical hydrophobic ‘knobs-into-holes’ packing required for dimerization. Second, this interfacial collapse corresponds to a broader structural uncertainty and backbone instability, as indicated by a significant reduction in both the average pLDDT (from 62.22 to 49.36) and overall topological confidence (pTM dropped from 0.56 to 0.33). Together, these robust computational data firmly establish that R-EABR loses its ESCRT-recruiting function due to a complete collapse of the dimeric binding interface and global assembly, perfectly justifying its use as a rigorous sequence-specific negative control. Quantitative multimer and heptad parameters are summarized in Supplementary Tables S2 and S3.

For plasmid construction, we utilized the pcDNA3.1-signal-sfGFP-EABR backbone and successfully generated the aforementioned chimeric plasmids via restriction enzyme digestion and seamless cloning techniques. The ligation products were transformed into competent cells, and positive single colonies were verified by PCR amplification using specific primers. Agarose gel electrophoresis revealed the expected ~1500 bp target bands for all recombinant plasmids (Figure S4). Subsequent Sanger sequencing confirmed that all inserted sequences were 100% matched to the in silico designs without any frameshift mutations. These sequence-verified plasmids were then extracted at high purity for subsequent functional expression and validation in HEK293T cells.

### 3.3. Verification of EABR-Mediated CYP3A4 Expression and Specific Sorting into Extracellular Vesicles

To evaluate the regulatory role of the EABR motif on CYP3A4 expression and its vesicular secretion, we transfected HEK293T cells with various chimeric plasmids: N-EABR-CYP3A4, C-EABR-CYP3A4, wild-type CYP3A4 (WT-CYP3A4), and the reverse-insertion mutant (R-EABR-CYP3A4). At 48 h post-transfection, intracellular lysates and purified extracellular vesicles (EVs) from the conditioned media were collected for Western blot analysis. Because some constructs lacked the FLAG tag, a specific Anti-CYP3A4 primary antibody was utilized uniformly for detection.

**Figure 3:**
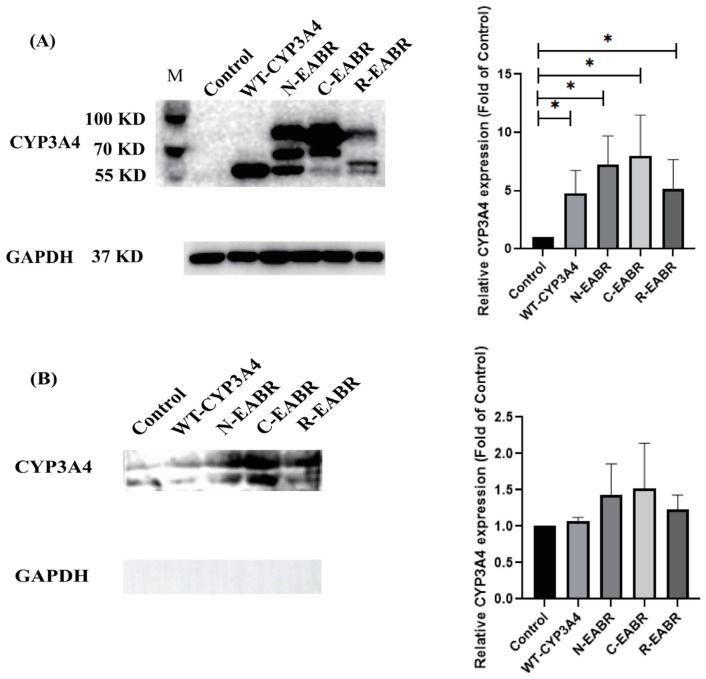
Densitometric quantification of CYP3A4 protein expression in 24-well plate culture. The band intensities of CYP3A4 were quantified using ImageJ software, normalized to the internal control (GAPDH), and are presented as fold changes relative to the Control group. Data are presented as the mean ± SD from three independent biological replicates (*n* = 3). Statistical significance was determined using Student’s *t*-test. * *p* < 0.05, (**A**) Intracellular fraction; (**B**) crude extracellular fraction.

As shown in Figure 3, WT-CYP3A4 exhibited a specific band at approximately 58 kDa. As expected, the molecular weights of all EABR-fused recombinant proteins migrated to approximately 70 kDa due to the insertion of the ~12 kDa EABR-containing polypeptide sequence. In the intracellular fraction (Figure 3A), robust expression of CYP3A4 was successfully detected across all experimental groups. The double bands around 70 kDa observed in the N-EABR and C-EABR groups likely represent different post-translational modification states (e.g., glycosylation or phosphorylation) acquired during the ESCRT-mediated secretory pathway. In contrast, the R-EABR mutant presents as a single unmodified band, likely because its disrupted spatial structure prevents efficient ESCRT recognition and entry into this normal sorting pathway. The additional 55 kDa band in the N-EABR group likely arises from partial intracellular proteolytic cleavage of the N-terminal EABR tag, thereby exposing the WT-like CYP3A4 core. It should be noted that the absolute yield of secreted EVs from the 24-well plate culture was intrinsically low. Nevertheless, in the crude extracellular fraction (Figure 3B), the groups with forward-inserted EABR (N-EABR and C-EABR) still exhibited a markedly enhanced CYP3A4 abundance compared to the WT construct.

Crucially, the R-EABR mutant, which possesses a disrupted spatial structure, failed to efficiently drive the exocytosis of CYP3A4. This contrast compellingly demonstrates that while the EABR motif significantly enhances the overall expression of CYP3A4, its mediated exocytosis is strictly dependent on its specific spatial sequence structure, consistent with the canonical parallel coiled-coil recognition required for ESCRT core component recruitment.

**Figure 4:**
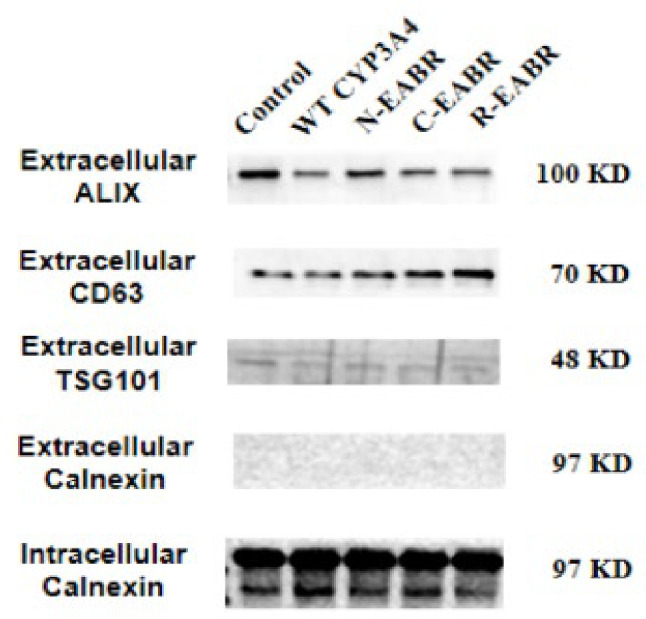
Protein expression levels of exosomal markers CD63, ALIX, and TSG101 in the extracellular fraction (crude supernatant), and the ER marker calnexin in the intracellular fraction.

To unequivocally confirm that the CYP3A4 detected in the supernatant was encapsulated within intact EVs rather than originating from free proteins released by cell lysis, we assessed canonical EV markers in the extracellular fractions (Figure 4). To obtain the crude supernatant, the conditioned medium was clarified by centrifugation at 15,000 rpm (equivalent to 21,130× *g*) for 5 min, bypassing the subsequent ultracentrifugation step. Calnexin, an endoplasmic reticulum-resident membrane protein, was exclusively detected in the intracellular lysate and was completely absent in the EV fractions, effectively ruling out cellular debris contamination. Conversely, classic EV markers, including CD63, ALIX, and TSG101, were significantly enriched in the extracellular components. These results comply with the quality control standards of the International Society for Extracellular Vesicles (ISEV) and mechanistically validate that recombinant CYP3A4 is specifically sorted and packaged into EVs for secretion by hijacking the host ESCRT pathway.

### 3.4. In Vitro Catalytic Activity and Transmembrane Kinetic Analysis of EV-Encapsulated CYP3A4

Following the confirmation of EABR-driven vesicular secretion, we evaluated the functional activity of the recombinant CYP3A4 variants using midazolam (MDZ), a classic and highly specific probe substrate for CYP3A4. At 48 h post-transfection, HEK293T cells were incubated with MDZ in the conditioned medium for 3 h. The generation of the primary metabolite, 1-OH-MDZ, in both the intracellular and extracellular compartments was relatively quantified via LC-MS/MS, using loratadine as an internal standard to calculate the peak area ratio (A1−OH−MDZ/ALoratadine).

**Figure 5:**
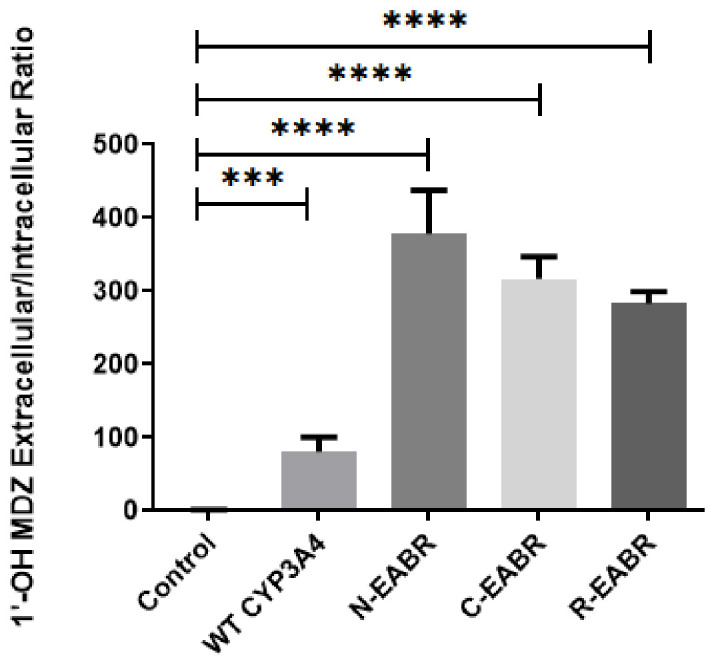
In vitro catalytic activity and specific vesicular secretion efficiency of different CYP3A4 constructs. The extracellular-to-intracellular ratio of 1′-OH-MDZ production, representing the specific efflux efficiency. The ratio was calculated to exclude the confounding effect of overall expression variations among different constructs. Data are presented as the mean ± SD derived from three independent biological replicates (*n* = 3). Statistical significance was determined using Student’s *t*-test, *** *p* < 0.001, **** *p* < 0.0001.

As illustrated in Figure 5, all CYP3A4-expressing groups exhibited significant 1-OH-MDZ production compared to the untransfected blank control. The relative metabolic activity followed the order C-EABR > N-EABR > WT-CYP3A4 > R-EABR. This apparent activity trend aligns closely with the intracellular expression abundance of the recombinant proteins (Figure 3), indicating that the EABR fusion does not perturb the native folding of the CYP3A4 catalytic domain; instead, it significantly enhances the total catalytic efficiency of the system by increasing the absolute abundance of the functional enzyme.

To evaluate the specific vesicular secretion efficiency of different CYP3A4 constructs, we measured the catalytic production of 1′-OH-MDZ in both extracellular and intracellular fractions. To eliminate the confounding effect of overall expression variations and accurately reflect the actual efflux efficiency, we calculated the extracellular-to-intracellular ratio of the metabolite (Figure 5). Our analysis reveals that the specific efflux ratios for both the N-EABR and C-EABR groups were higher compared to the wild-type CYP3A4 group. These results demonstrate that the EABR motif actively facilitates the targeted vesicular secretion of CYP3A4, indicating that the enhanced extracellular signal is not merely a byproduct of increased overall protein expression.

### 3.5. Biophysical Characterization and Enzymatic Functional Validation of EABR-Derived EVs

Our previous results indicated that recombinant CYP3A4 may be secreted extracellularly via a vesicular pathway. To definitively elucidate the physical properties of these recombinant vesicles and the functional state of the encapsulated CYP3A4, we scaled up the expression of the EABR-empty control, N-EABR-CYP3A4, and C-EABR-CYP3A4 constructs and purified extracellular vesicles (EVs) from the conditioned media using ultracentrifugation.

**Figure 6:**
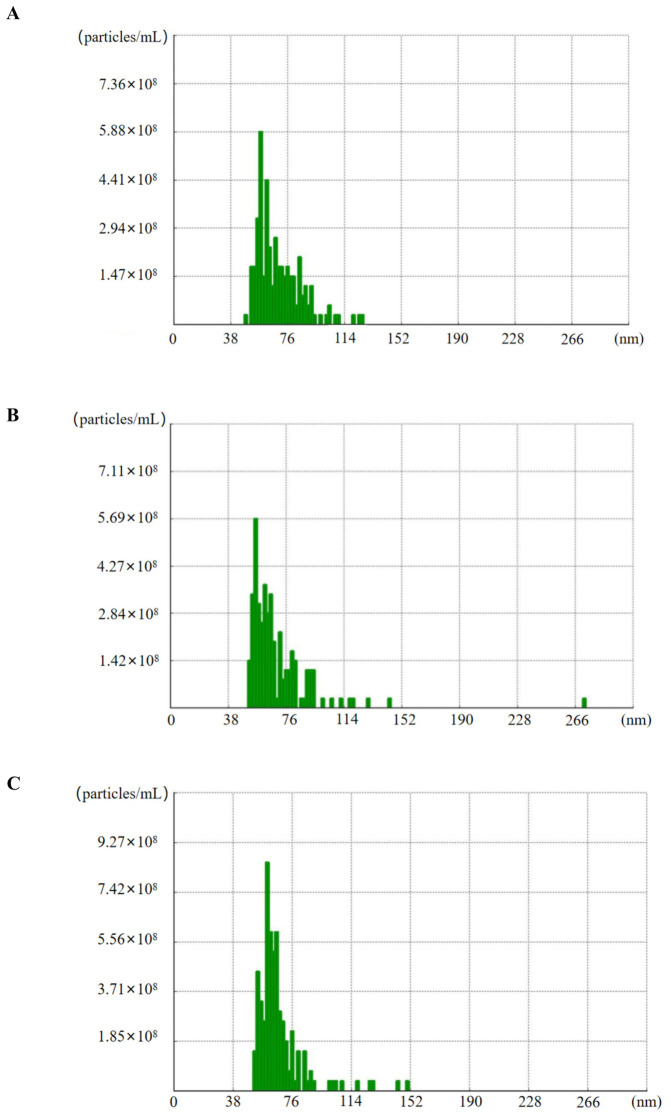
Analysis of EVs with resistive pulse sensing (RPS). (**A**) Control; (**B**) N-EABR; (**C**) C-EABR.

**Figure 7:**
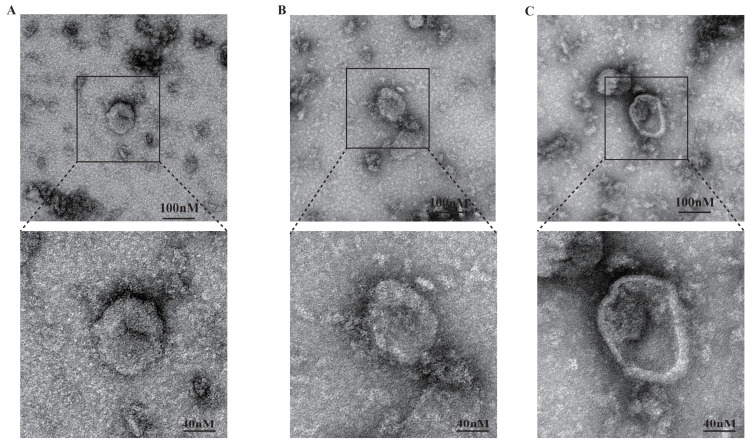
EVs imaged by Transmission Electron Microscope (TEM). (**A**) Control; (**B**) N-EABR; (**C**) C-EABR.

We first characterized the hydrodynamic size of the purified EVs using resistive pulse sensing (RPS). The results demonstrated that EVs from all three groups were isolated at high purity, exhibiting a typical unimodal size distribution with a major peak between 50 and 90 nm (Figure 6). This size range is highly consistent with the established physical profiles of small EVs (exosomes) generated via endosomal or plasma membrane budding pathways. The average nanoparticle concentrations of the extracellular vesicles were determined to be 1.29 × 10^11^, 1.27 × 10^11^, and 1.63 × 10^11^ particles/mL for the Control, N-EABR, and C-EABR groups, respectively. Furthermore, by calculating the particle-to-protein ratios to evaluate the enrichment quality, the EV purities were determined to be 3.00 × 10^10^ particles/mg, 4.58 × 10^10^ particles/mg, and 5.21 × 10^10^ particles/mg for the Control, N-EABR, and C-EABR groups, respectively. Furthermore, morphological examination via transmission electron microscopy (TEM) revealed that the purified particles exhibited the characteristic “cup-shaped” morphology featuring a depressed lipid bilayer commonly observed in negative-stained EV preparations (Figure 7), visually confirming the successful isolation of intact EVs.

**Figure 8:**
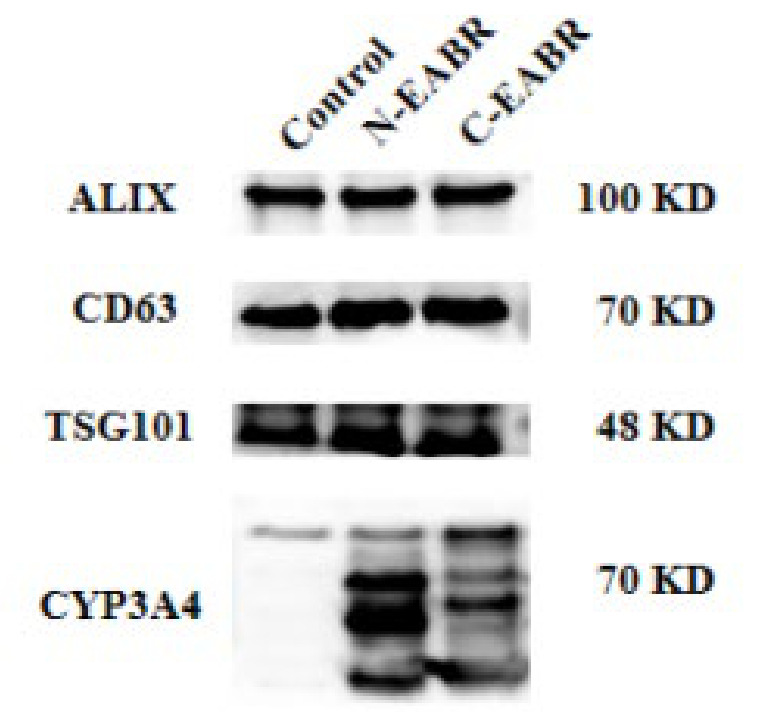
Expression of the classical extracellular vesicle (EV) markers ALIX, CD63, TSG101, and CYP3A4 protein. Extracellular vesicle (EV) pellets purified from conditioned media via 150,000× *g* ultracentrifugation after 96 h of cell culture in 10 cm dishes.

Following biophysical validation, we conducted an in-depth analysis of the EV composition and function. Western blot analysis of the EV pellets not only confirmed the presence of the canonical tetraspanin EV marker CD63 but also revealed the enrichment of TSG101 and ALIX (Figure 8). This molecular signature aligns perfectly with recent mechanistic studies demonstrating that the CEP55-EABR motif acts as a molecular hinge to directly recruit host ALIX and the ESCRT-I core protein (TSG101) with high affinity, thereby potently driving the assembly, membrane fission, and release of target protein-laden vesicles. Notably, robust CYP3A4 expression was exclusively detected in the EVs derived from the N-EABR and C-EABR groups, whereas it was entirely absent from the EABR-only control EVs, confirming that the vesicular export of CYP3A4 is strictly target-specific.

**Figure 9:**
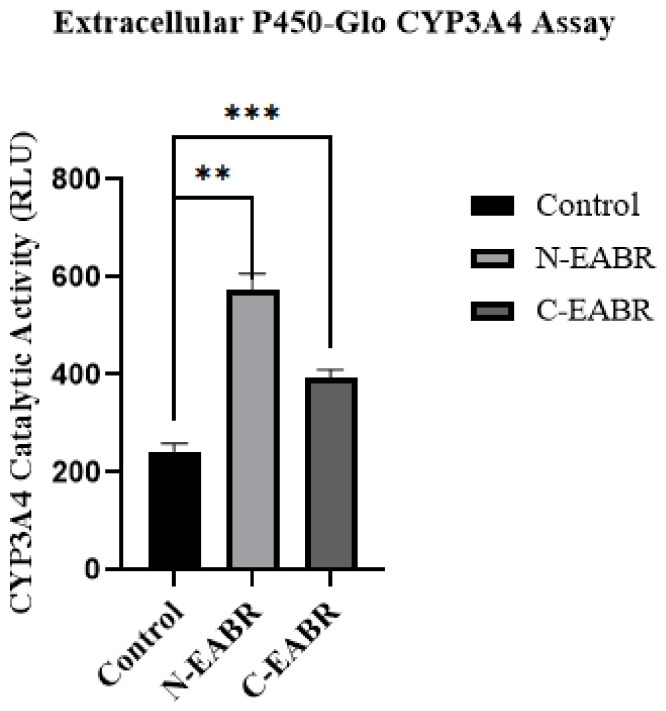
In vitro catalytic activity of CYP3A4 encapsulated in purified EVs. Purified EVs, prepared as described in Figure 8, were incubated with Luciferin-IPA, a specific pro-luminescent probe cleaved by CYP3A4 to release D-luciferin. This D-luciferin reacts with excess exogenous luciferase in the detection reagent to produce light. Thus, the resulting relative luminescence units (RLU) directly and exclusively quantify CYP3A4 catalytic activity, not endogenous luciferase expression. Data are presented as mean ± SD from three independent biological replicates (*n* = 3). Statistical significance was determined using Student’s *t*-test. ** *p* < 0.01, *** *p* < 0.001.

Finally, we evaluated the in vitro metabolic activity of the purified EV-CYP3A4 complexes. Compared to the EABR control EVs lacking the target enzyme, both the N-EABR and C-EABR vesicle groups exhibited significantly enhanced and specific CYP3A4 catalytic turnover (Figure 9). As a highly hydrophobic membrane protein, CYP3A4 relies heavily on an intact lipid bilayer environment to maintain its complex allosteric conformations and facilitate the partitioning of lipophilic substrates. Here, the native EV membrane generated via EABR-mediated budding not only circumvents the enzyme inactivation typically caused by detergent-based purification, but also provides an optimal biomimetic lipid microenvironment. Consequently, the EV-encapsulated CYP3A4 retains robust drug-metabolizing capabilities even in a cell-free system.

In addition, we simultaneously detected the expression and activity of CYP3A4 in cells, and the results were consistent with those of exosome detection: compared with the Control group, the forward insertion of EABR (both N-EABR and C-EABR) could significantly upregulate its expression and enhance its metabolic activity (Supplemental Figures S5 and S6).

## 4. Discussion

In this study, we successfully established a novel membrane protein engineering platform utilizing the EABR motif to drive the targeted vesicular secretion of Cytochrome P450 3A4. Previous structural studies have demonstrated that the EABR motif forms a noncanonical parallel coiled-coil structure to specifically recruit TSG101 of the ESCRT-I complex and the accessory protein ALIX, thereby driving membrane fission and promoting the overall biogenesis of extracellular vesicles [20]. This mechanism is also hijacked by enveloped viruses such as SARS-CoV-2 and HIV to facilitate budding [24]. Interestingly, our results indicate that the topological position of the EABR insertion significantly influences the balance between catalytic fidelity and sorting efficiency. The forward insertion is topologically more favorable for recruiting the ESCRT machinery on the cytosolic side, yielding enzyme-containing vesicles of higher abundance and purity.

It is also crucial to address the methodological considerations regarding EV characterization. While GAPDH is a common cytosolic control, it can be non-specifically encapsulated into EVs during robust membrane budding. To strictly comply with MISEV guidelines, we instead employed the endoplasmic reticulum-resident protein Calnexin as a definitive negative control. Its complete absence in our purified EVs confirms that EABR-mediated CYP3A4 secretion is a highly specific ESCRT-dependent sorting process, rather than non-specific cell lysis or organelle leakage.

Compared to commercially available CYP3A4 baculosomes traditionally produced in insect cells, our mammalian EV system offers distinct advantages. The HEK293T-derived EVs provide a more physiologically relevant lipid composition (e.g., appropriate cholesterol and phospholipid ratios), which is vital for the optimal thermal stability and substrate recognition of CYP3A4 [28]. Economically, our plasmid-based transient transfection system provides a highly scalable “plug-and-play” alternative that bypasses the time-consuming and expensive baculovirus packaging and amplification processes, thus holding great potential for industrial applications.

Despite these advantages, certain limitations must be acknowledged. First, to mitigate the potential co-isolation of non-membranous protein complexes inherent to ultracentrifugation, future studies should consider combining differential ultracentrifugation with size-exclusion chromatography (SEC) for optimal EV purity. Furthermore, although this proof-of-concept study successfully established an efficient EV-CYP3A4 secretion platform that preserves catalytic function within a native-like lipid microenvironment, the overall catalytic efficiency of the system may still be limited by the absence of an intrinsic redox electron transfer chain. In the native endoplasmic reticulum, CYP3A4 activity is highly dependent on dynamic coupling with cytochrome P450 reductase (CPR) [29]. Currently, to perform in vitro metabolism assays using isolated EVs with midazolam (MDZ) as a substrate, the EV-CYP3A4 complex must be co-incubated with exogenous CPR and an NADPH regeneration system in solution to successfully generate the 1′-OH-MDZ metabolite. To address this limitation and fully exploit this novel platform, future studies will employ multiple tagging systems (e.g., EABR and EPM) to achieve precisely controlled co-packaging of CYP3A4, CPR, and potentially cytochrome b5 within the same EVs. Ultimately, this approach will facilitate the creation of a fully functional, self-sufficient P450 biomimetic bioreactor in vitro that eliminates the requirement for exogenous helper proteins. Based on this standardized bioreactor, our next phase of research will focus on obtaining absolute enzymatic kinetic parameters (Km and Vmax) and conducting large-scale drug–drug interaction (DDI) inhibition assays for high-throughput pharmacological and toxicological screening.

## 5. Conclusions

Collectively, our findings demonstrate that the forward CEP55-EABR motif serves as a robust and specific molecular tool for facilitating the vesicular packaging and secretion of membrane-anchored CYP3A4. By leveraging the endogenous ESCRT machinery, this approach circumvents the limitations of traditional purification methods that compromise protein structure and function, thereby maintaining the natural activity of CYP3A4 in a lipid-rich EV microenvironment. Beyond providing a novel technical solution for stable membrane protein expression, this ESCRT-mediated engineering strategy paves the way for advancing in vitro drug metabolism research and optimizing high-throughput drug screening technologies, holding substantial promise for translational applications in pharmaceutical development and membrane protein engineering.

## Data Availability

All data used to support this study are available from the corresponding author upon request. Reagents and all other data are also available from the corresponding author upon reasonable request. The datasets have been submitted as Supplemental Materials.

## Supplementary Materials

The following supporting information can be downloaded at https://www.mdpi.com/article/10.3390/biomedicines14061299/s1: Table S1: List of primer sequences required for plasmid construction; Table S2: Quantitative parameters derived from single-chain AlphaFold predictions and sequence-based heptad fingerprint analysis; Table S3: Top-line multimer metrics comparison between the forward (EPM-EABR) and reversed (EABR-EPM) homodimers predicted by AlphaFold. Figure S1: Amino acid sequence diagram of CYP3A4 with transmembrane helices marked in orange; Figure S2: Amino acid sequence diagram of CYP3A4 with transmembrane helices marked in orange; Figure S3: Computational structural validation of the wild-type EABR and R-EABR constructs using AlphaFold-Multimer and sequence-based fingerprint analyses; Figure S4: Agarose gel electrophoresis of bacterial liquid PCR for CYP3A4 recombinant plasmid identification; Figure S5: Western blot analysis of intracellular CYP3A4 expression; Figure S6: Intracellular P450-Glo CYP3A4 assay.
