# Supplementary material for: A Novel Technology Platform for Extracellular Vesicle-Targeted Expression of Drug-Metabolizing Enzymes: Driving CYP3A4 Expression and Secretion via the EABR Motif
Source: Biomedicines. 2026 Jun 8;14(6):1299. doi: 10.3390/biomedicines14061299 (PMC13296741; doi:10.3390/biomedicines14061299)
Supplement: Supplementary file 1 [file biomedicines-14-01299-s001.zip › supplemental figures.pdf]

Supplemental Table S1 List of primer sequences required for plasmid construction

**Primers**	**Sequences（5'-3'）**
5-EABR	GACCCAAGCTGGCTAGCGCCACCATGTTCAACTCCTCCATCAATAACATCC
3-EABR	CGCCGCTTCCGCCGGGCAGGGAGTGGGCGGCGGT
5-EPM	CCTGCCCGGCGGAAGCGGCGCTCTGCCTGGAAAT
3-EPM	GTCATCATCGTCCTTGTAGTCGGATCCGGAGCCGTAGGGGCTGGAGCTTGTT
5N-CYP3A4	CCTACGGCTCCGGATCCAGAATGGCTCTCATCCCAGACTTG
3N-CYP3A4	GGGTTTAAACGGGCCCGATCCTTACTTGTCATCATCGTCCT
5C-CYP3A4	AGGCCACCATGGGATCCAGAATGGCTCTCATCCCAGACTTG
3C-CYP3A4	CTTCCGCCGCCGCTAGCGGCTCCACTTACGGTGCCATCCC
5R-CYP3A4	ATGATGACAAGAAGCTTAGAATGGCTCTCATCCCAGACTTG
3R-CYP3A4	TGCAGTACCAGCTCGAGttaGGCTCCACTTACGGTGCCATCCC

Supplemental Table S2 Quantitative parameters derived from single-chain AlphaFold predictions and sequence-based heptad fingerprint analysis.

label	length	mean_pLDDT	median_pLDDT	best_register	a/d_hydrophobic_enrichment	e/g_abs_charge_mean	a/d_fraction
EABR	109	73.92908256880735	76.62	1	1.8775847808105874	0.26129032258064516	0.28440366972477066
REABR	109	73.02412844036698	70.56	6	1.8775847808105872	0.26129032258064516	0.28440366972477066

Supplemental Table S3 Top-line multimer metrics comparison between the forward (EPM-EABR) and reversed (EABR-EPM) homodimers predicted by AlphaFold.

Metric	Forward Homodimer (EPM-EABR)	Reversed Homodimer (EABR-EPM)	Interpretation
ipTM	0.56	0.21	Drop indicates loss of functional dimeric interface
pTM	0.56	0.33	Indicates reduced global topological confidence
ipSAE	0.565	0.012	Collapse reflects loss of interface specificity
Mean PAE	18.22	22.86	Increase indicates uncertain relative chain placement
Avg pLDDT	62.22	49.36	Decrease reflects broader structural uncertainty

Supplemental Figures

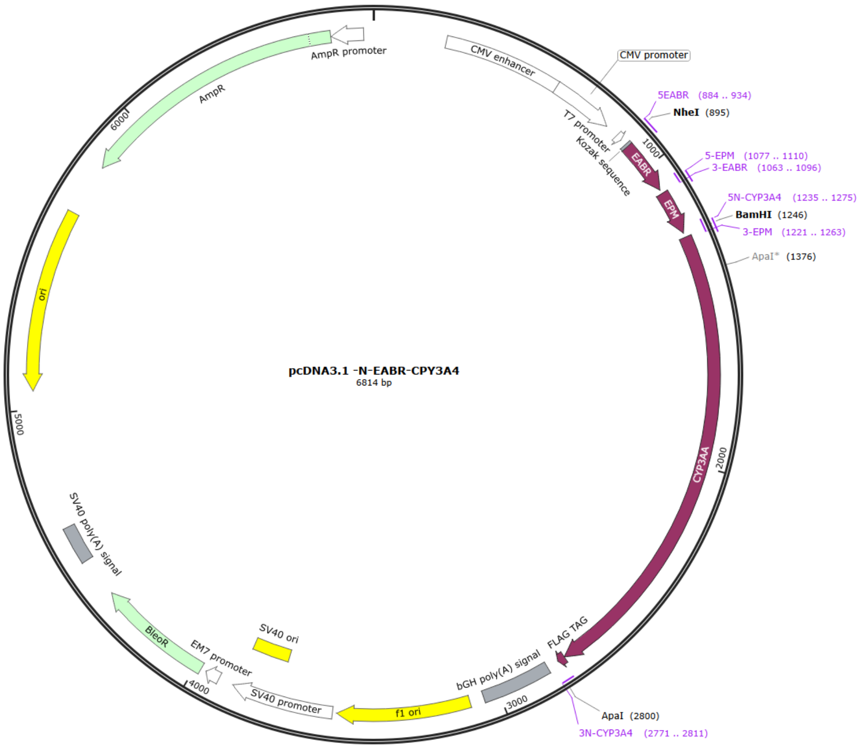

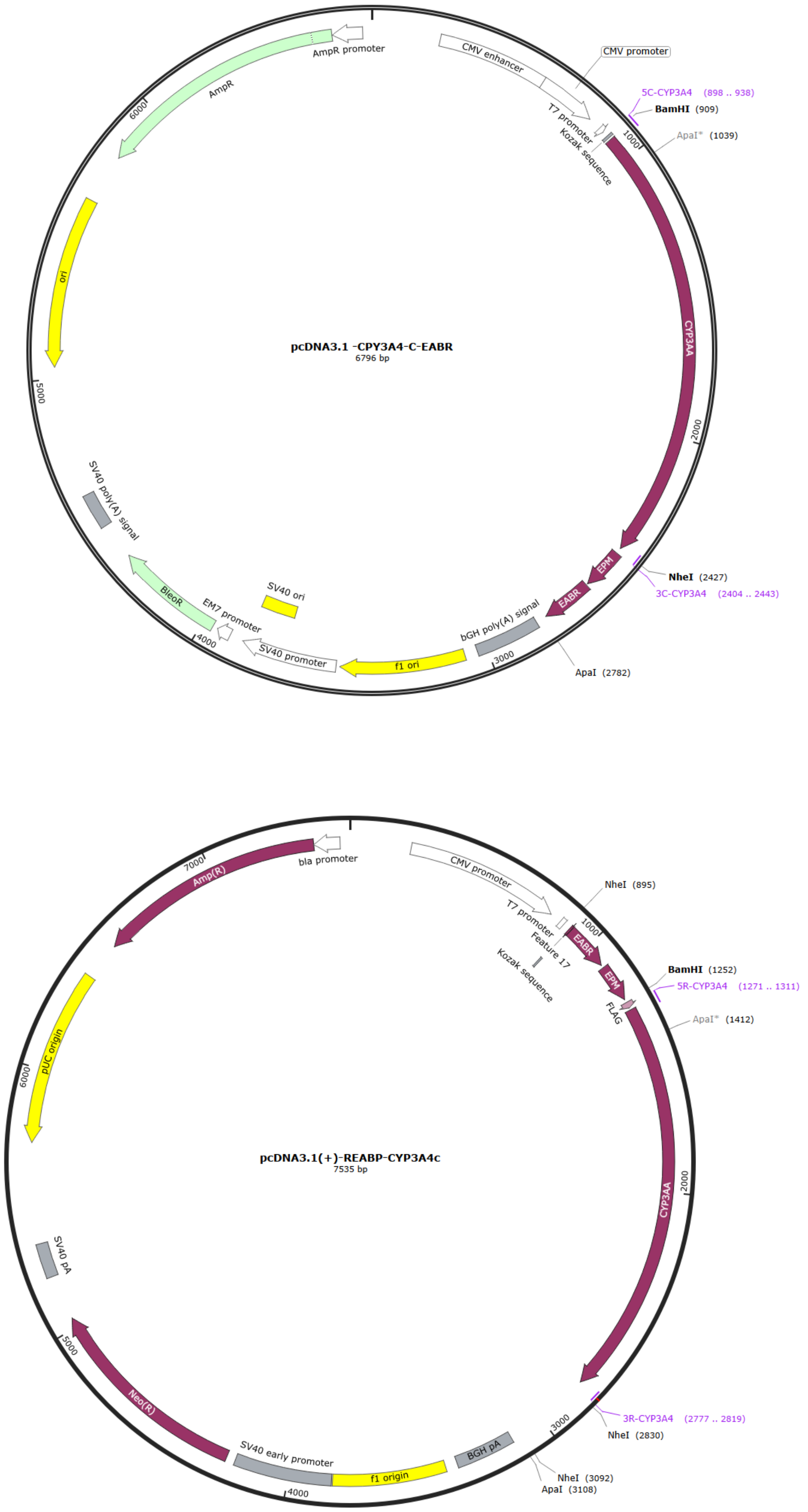

Figure S1. The corresponding schematic vector maps, (A) N-EABR; (B) C-EABR; (C)R-EABR

1 MALIPDLAME TWLLLAVSLV LLYLYGTHSH GLFKKLGIPG PTPLPFLGNI LSYHKGFCMF
61 DMECHKKYGK VWGFYDGQQP VLAITDPDMI KTVLVKECYS VFTNRRPFGP VGFMKSAISI
121 AEDEEWKRLR SLLSPTFTSG KLKEMVPIIA QYGDVLVRNL RREAETGKPV TLKDVFGAYS
181 MDVITSTSFG VNIDSLNNPQ DPFVENTKKL LRFDFLDPFF LSITVFPFLI PILEVLNICV
241 FPREVTNFLR KSVKRMKESR LEDTQKHRVD FLQLMIDSQN SKETESHKAL SDLELVAQSI
301 IFIFAGYETT SSVLSFIMYE LATHPDVQQK LQEEIDAVLP NKAPPTYDTV LQMEYLDMVV
361 NETLRLFPIA MRLERVCKKD VEINGMFIPK GVVVMIPSYA LHRDPKYWTE PEKFLPERFS
421 KKNKDNIDPY IYTPFGSGPR NCIGMRFALM NMKLALIRVL QNFSFKPCKE TQIPLKLSLG
481 GLLQPEKPVV LKVESRDGTV SGA*

Figure S2. Amino acid sequence diagram of CYP3A4 with transmembrane helices marked in orange.

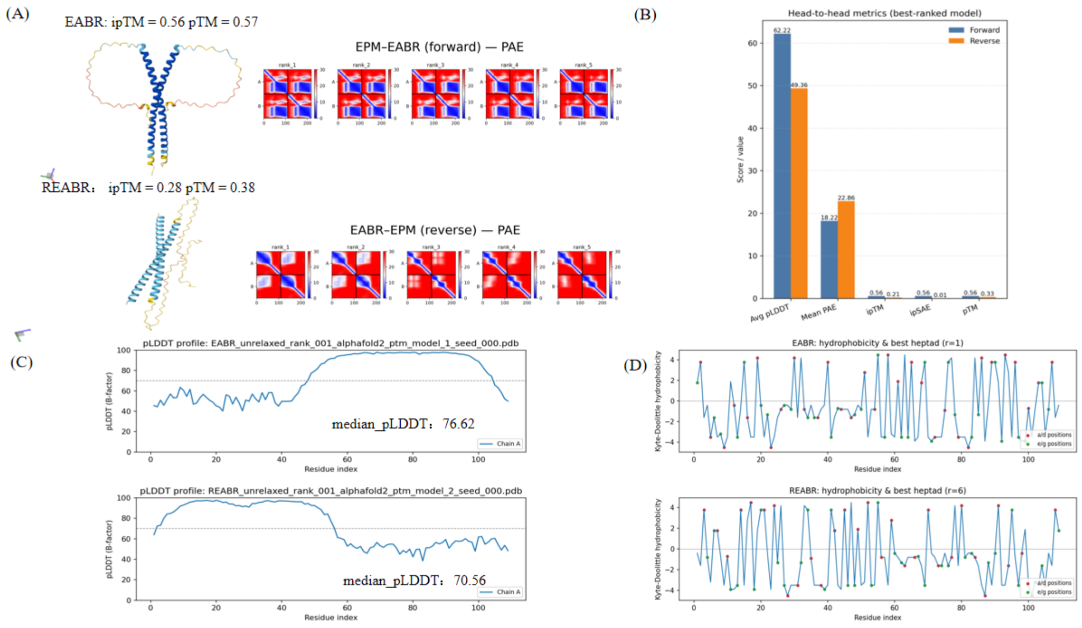

Figure S3. Computational structural validation of the wild-type EABR and R-EABR constructs using AlphaFold-Multimer and sequence-based fingerprint analyses. (A) 3D structural predictions and the corresponding Predicted Aligned Error (PAE) heatmaps generated by the AlphaFold 3 server. Left: 3D models and top-line confidence scores. The wild-type EABR forms a highly stable parallel coiled-coil homodimer (ipTM = 0.56, pTM = 0.57), whereas the R-EABR completely fails to dimerize (ipTM = 0.28, pTM = 0.38). Right: The corresponding PAE heatmaps. The forward EPM-EABR chimera shows a compact, low-error interaction block at the dimeric interface, which is uniformly elevated and completely abolished in the reversed sequence, indicating a complete loss of inter-chain geometry. (B) Grouped bar chart comparing key multimer confidence metrics (Avg pLDDT, Mean PAE, ipTM, ipSAE, and pTM) between the forward and reversed constructs. The data demonstrate a catastrophic drop in interface confidence and specificity (e.g., ipTM drops from 0.56 to 0.21; ipSAE from 0.565 to 0.012) upon sequence reversal. (C) Single-chain per-residue pLDDT profiles. The reversed sequence exhibits a more heterogeneous local confidence distribution and a lower median pLDDT (~70.6 vs. ~76.6), indicating decreased single-chain backbone stability. (D) Sequence-based heptad fingerprint analysis. The hydrophobicity scanning plots demonstrate that sequence reversal causes a massive shift in the optimal heptad phase (from r=1 to r=6), mathematically abolishing the canonical hydrophobic "knobs-into-holes" packing and charge complementarity required for successful coiled-coil dimerization.

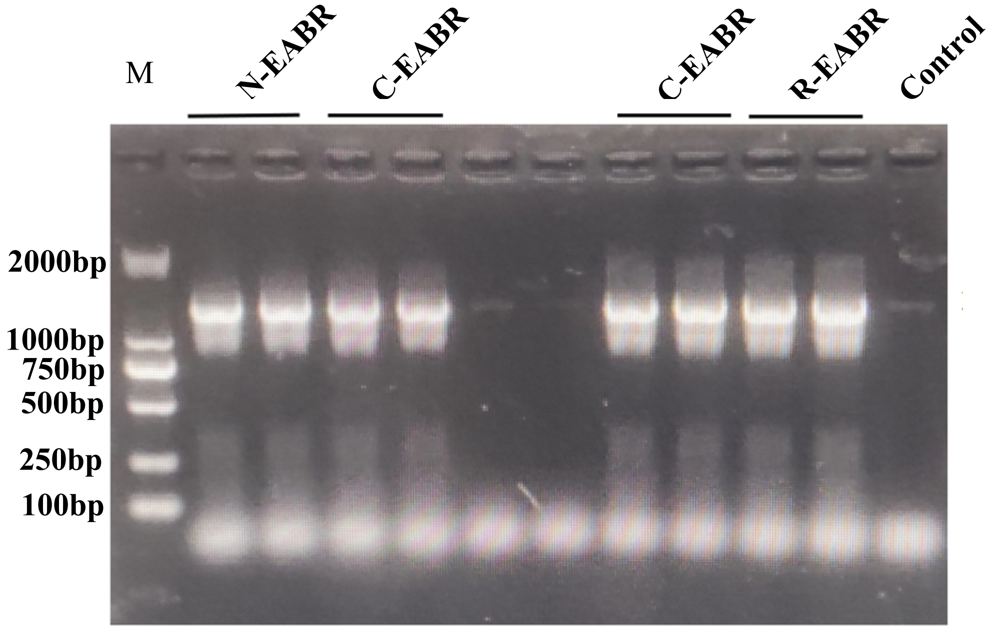

Figure S4. Agarose gel electrophoresis of bacterial liquid PCR for CYP3A4 recombinant plasmid identification.

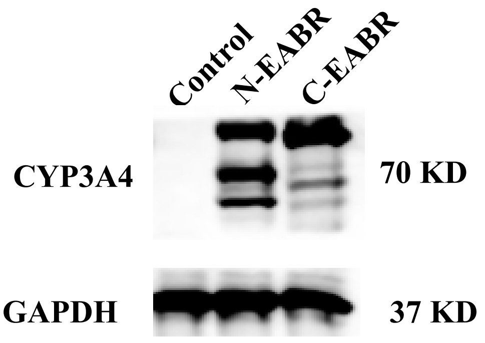

Figure S5. Western blot analysis of intracellular CYP3A4 expression.

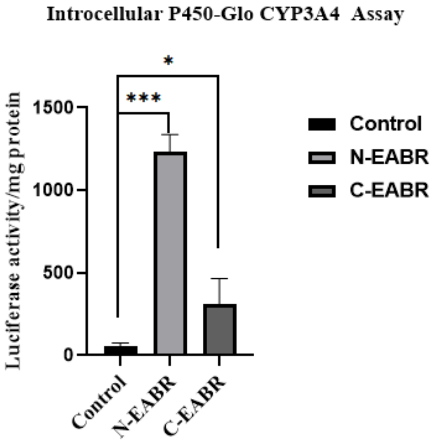

Figure S6. Intracellular P450-Glo CYP3A4 assay.
